# Terpenoid-Mediated Targeting of STAT3 Signaling in Cancer: An Overview of Preclinical Studies

**DOI:** 10.3390/biom14020200

**Published:** 2024-02-07

**Authors:** Fahad Khan, Pratibha Pandey, Meenakshi Verma, Tarun Kumar Upadhyay

**Affiliations:** 1 Center for Global Health Research, Saveetha Medical College and Hospitals, Saveetha Institute of Medical and Technical Sciences, Saveetha University, Chennai 602105, India; fahadintegralian@gmail.com; 2 University Centre for Research and Development, Chandigarh University, Gharuan, Mohali 140413, India; 3 Department of Chemistry, University Institute of Sciences, Chandigarh University, Gharuan, Mohali 140413, India; 4 Department of Biotechnology, Parul Institute of Applied Sciences and Research and Development Cell, Parul University, Vadodara 391760, India; tarun_bioinfo@yahoo.co.in

**Keywords:** terpenoids, STAT3, cancer therapy, natural products, signaling

## Abstract

Cancer has become one of the most multifaceted and widespread illnesses affecting human health, causing substantial mortality at an alarming rate. After cardiovascular problems, the condition has a high occurrence rate and ranks second in terms of mortality. The development of new drugs has been facilitated by increased research and a deeper understanding of the mechanisms behind the emergence and advancement of the disease. Numerous preclinical and clinical studies have repeatedly demonstrated the protective effects of natural terpenoids against a range of malignancies. Numerous potential bioactive terpenoids have been investigated in natural sources for their chemopreventive and chemoprotective properties. In practically all body cells, the signaling molecule referred to as signal transducer and activator of transcription 3 (STAT3) is widely expressed. Numerous studies have demonstrated that STAT3 regulates its downstream target genes, including *Bcl-2*, *Bcl-xL*, *cyclin D1*, *c-Myc*, and *survivin*, to promote the growth of cells, differentiation, cell cycle progression, angiogenesis, and immune suppression in addition to chemotherapy resistance. Researchers viewed STAT3 as a primary target for cancer therapy because of its crucial involvement in cancer formation. This therapy primarily focuses on directly and indirectly preventing the expression of STAT3 in tumor cells. By explicitly targeting STAT3 in both in vitro and in vivo settings, it has been possible to explain the protective effect of terpenoids against malignant cells. In this study, we provide a complete overview of STAT3 signal transduction processes, the involvement of STAT3 in carcinogenesis, and mechanisms related to STAT3 persistent activation. The article also thoroughly summarizes the inhibition of STAT3 signaling by certain terpenoid phytochemicals, which have demonstrated strong efficacy in several preclinical cancer models.

## 1. Introduction

The process of cancer advancement is intricate and multifaceted, starting with aberrant cells that have the potential to become malignant or neoplastic. Subsequently, the tumor grows, stromal invasion occurs, and metastasis occurs. This occurrence depends not only on tumor-intrinsic effects but also on the tumor microenvironment, which includes surrounding and supporting stroma, humoral factors, various immune system effectors, and vasculature [1,2]. The discovery of the Janus kinase (JAK)-signal transducer and activator of transcription (STAT) pathway occurred initially within the framework of downstream signaling mediated by interferon-α (IFNα), interferon-γ (IFNγ), and interleukin-6 (IL-6) [3,4]. Based on available data, STAT3 and STAT5, two of the seven members of the STAT protein family, are crucial for advancing cancer. They are essential transcription factors that control the expression of a wide variety of genes [5,6], contributing to tumor growth. In addition, they are vital for transducing signals from many receptor and non-receptor tyrosine kinases that are commonly active in cancer cells. Although STAT3 and STAT5 both play a role in the survival and multiplication of tumor cells, STAT3 stands out as a potentially effective target for cancer treatment because it served as an important player in the recruitment of stromal cells such as immune cells to the tumor microenvironment, which supports the growth of tumors [7,8].

In addition to being an oncogene and transcription activator, STAT3 is essential for tumor cell proliferation, invasion, and migration. It can induce the epithelial–mesenchymal transition (EMT), regulate the tumor microenvironment, and support cancer stem cell (CSC) self-renewal and differentiation, all of which are beneficial to the advancement of cancer [9,10]. STAT3 is commonly found with stimulated activity in most human carcinoma cell lines and tumor tissues. Previous research has demonstrated that STAT3 can also control the expression of genes by epigenetic alteration. For example, unphosphorylated STAT31 can control the architecture of the chromatin, and acetylated STAT3 can contribute to the DNA methylation that silences tumor-suppressor genes [11]. Numerous findings have demonstrated the pivotal function of STAT3 in developing cancer, rendering it a prime candidate for cancer treatment [12].

Recently, numerous unforeseen functions of the JAK-STAT3 pathway in cancer have surfaced, along with the fundamental processes via which this route is triggered and carries out its carcinogen-promoting impacts. Historically, cytokines and growth factors were assumed to be the primary activators of the JAK-STAT3 pathway. Toll-like receptors (TLRs) such as TLR9 and TLR4 have been found in several recent investigations to be significant activators of the JAK–STAT3 pathway [13,14]. In turn, STAT3 stimulates the production of specific TLRs in cancerous cells, accelerating the growth of tumors [15,16].

Several studies have found a link between nutrition and cancer therapeutic efficacy. Using phytochemicals from dietary and medicinal plants appropriately has been demonstrated to reduce cancer mortality by affecting the activation of many carcinogenic molecules [17,18]. An increasingly popular and feasible method of lessening the effects of cancer is natural cancer chemoprevention, which focuses on phytochemicals, vitamins, and minerals as a safe and affordable alternative to anticancer medication. Unlike pharmaceutical drugs, which are mono-target molecules, herbal treatments contain multitarget substances, meaning they can regulate the start and spread of cancer [19,20]. Recent research on these natural chemicals has revealed that they target several cellular signaling pathways, including NF-kB, MAPK, Wnt, Akt, Notch, p53, AR, and ER pathways, to demonstrate their multifaceted impact on cancer cells. This implies that the prevention and treatment of tumor formation may benefit from using these natural substances, either on their own or in conjunction with traditional therapeutic drugs [21]. Terpenoids are the largest class of natural compounds and have been shown to have about 25,000 different chemical structures. They have potential uses in the chemical, fragrance, medicinal, and flavoring industries [22]. Terpenoids are classified into subclasses based on their structures: monoterpenoids, sesquiterpenoids, diterpenoids, triterpenoids, and tetraterpenoids. Terpenoids have garnered significant attention due to their potential health advantages, including the possibility of having anticancer properties and other relevant pharmacological activities [23]. This review article intends to investigate numerous naturally occurring terpenoids that can reduce the JAK/STAT signaling pathway, thereby decreasing cancer cell development caused by abnormal JAK/STAT signaling. We have compiled and evaluated the most recent studies to find putative therapeutic and chemopreventive terpenoids with known molecular targets.

## 2. Terpenoid-Mediated Cancer Chemoprevention

Several experimental findings have suggested that a diet high in fruits and vegetables was frequently linked to lower cancer risk. There have been investigations of effective phytochemicals inhibiting cancer signaling pathways. Consequently, the scientific and medical communities are very interested in the bioactivities of natural phytochemicals and their potential to prevent cancer [24,25]. A wide range of potential chemopreventive bioactives is being studied. The term “chemoprevention” was initially used by Sporn et al. to describe the use of natural or synthetic chemicals [26]. According to a recently developed definition, chemopreventive agents can inhibit the molecular pathways contributing to the genesis and spread of cancer. A potential link between dietary ingredients and carcinogenesis has been suggested by the diverse eating habits that have been increasingly responsible for either lower or greater incidences of cancer development in ethnic groups in various nations [27]. Cancers of the breast, prostate, and gastrointestinal system, for example, have been observed to be less common in the Asian population. Drug research can benefit from novel approaches provided by natural ingredients [28]. One of the most practical methods of controlling cancer may be phytochemicals like terpenoids through cancer chemoprevention. Terpenoids (also referred to as terpenes) are easily derived from vegetables, fruits, spices, teas, herbs, and medicinal plants and have been shown to reduce experimental carcinogenesis in several organs in preclinical models. Terpenoids are categorized into monoterpenes (C10), sesquiterpenes (C15), diterpenes (C20), sesterterpenes (C25), triterpenes (C30), tetraterpenes (C40), and polyterpenes based on the number of building units. Terpenoids, sometimes referred to as isoprenoids, are a family of naturally occurring chemicals derived from plants that are thought to be the most diversified and have a variety of crucial physiological roles [29,30]. Currently, terpenoids have more than 25,000 different chemical structures and potential uses in the chemical and fragrance industries. According to recent reports, the mechanisms underlying terpenoids’ potential to prevent cancer may involve a combination of immune-boosting, anti-inflammatory, antioxidant, and hormone modulation effects, as well as have an impact on drug-metabolizing enzyme expression, cell cycle progression and differentiation, induction of apoptosis, and suppression of proliferation and angiogenesis [31]. These mechanisms would therefore play a role in the initial and secondary modification stages of cancer development. Even though terpenes as dietary supplements have demonstrated enormous promise for cancer prevention in the past ten years, more thorough preclinical and translational research is still required [32]. We have thus investigated the chemopreventive mechanism of this class of natural chemicals by focusing on the targets of the STAT3 signaling pathway.

## 3. STAT3 Signaling Cascade and Tumorigenesis

**Figure 1:**
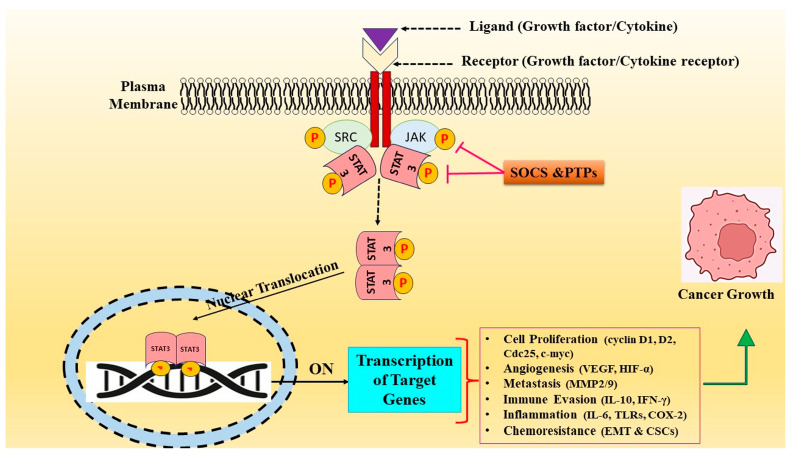
Various stimuli like growth factors and cytokines cause abnormal activation of the STAT3 signal transduction cascade. STAT3 phosphorylation causes nuclear localization, which stimulates STAT3 target genes implicated in cancer processes such as cell proliferation, angiogenesis, metastasis, invasion, and drug resistance.

The term “signal transducer and activator of transcription” comes from the cytoplasmic inducible transcription factors comprising signal transducer and activator of transcription (STAT) proteins. These proteins relay extracellular signals from the cell surface to the nucleus to express the target genes [33,34]. Since their discovery, the STAT protein family of transcription factors has been thoroughly researched in relation to cancer [35]. The seven members of the STAT family are STAT1, STAT2, STAT3, STAT4, STAT5a, STAT5b, and STAT6 [36,37]. The 750–850 amino acid range of these proteins varies, and their functional role is to mediate signals from growth factors (GFs) and cytokine receptors on the cell membrane to the nucleus [38]. Extracellular signaling proteins (growth factors and cytokines) frequently activate STAT3 signaling when they bind precisely to the appropriate receptors on the cell surface. Once activated by phosphorylation, STAT3 forms homodimers, moves into the nucleus, and attaches to DNA to regulate the expression of downstream genes, boosting cell proliferation and survival, invasion, angiogenesis, chemotherapeutic resistance, and immunological escape [39]. One important STAT3 upstream regulator is interleukin 6 (IL-6). When IL-6 binds to the IL-6 receptor (IL-6R), a heterohexameric complex is formed consisting of IL-6, IL-6R, and the membrane-spanning protein IL-6R subunit (gp130, also known as IL-6Rβ). Then, STAT3 and Janus kinases (JAKs) are recruited and activated. Afterward, pJAKs tyrosine phosphorylate the cytoplasmic portion of gp130 [40]. By identifying and binding to the phosphorylated tyrosine docking site on phosphorylated gp130, STAT3’s SH2 domain attracts STAT3 to the area around the active JAK enzyme. Following this, Tyr705 on STAT3 becomes phosphorylated, encouraging the SH2 domain to bind with Tyr705 to create a head-to-tail dimer. Following its dissociation from cell surface receptors and translocation into the nucleus, the pSTAT3 dimer controls the expression of several STAT3 target genes [41]. A positive feedback loop is created when cancer cells’ overactive STAT3 signaling pathway increases IL-6 synthesis. STAT3 can be activated by the non-receptor tyrosine kinase Src in addition to JAKs [42]. STAT3 can control the expression of genes linked to cancer development, and its chronic activation has been extensively studied in various human cancers [43] (Figure 1). Takeda et al. showed that selective loss of STAT3 in mice resulted in an early lethal phenotype during embryogenesis, and tissue-specific ablation showed decreased oncogenesis and increased inflammation [44]. Numerous genes that control antiapoptosis (*Bcl-2*, *Bcl-xL*, *survivin*, and *Mcl-1*) and cell cycle progression (*cyclin D1*, *c-Myc*, and *Pim1/2*), angiogenesis (*HGF*, *VEGF*, *bFGF*, *IL-17*, and *IL-23*), cell proliferation (*IL-6*, *CXCL12*, and *HIF-1α*), metastasis (*MMP-1/2/3/9*, *fascin*, *vimentin*, and *ICAM-1*), and immune suppression and inflammation (*IL-10*, *IL-23*, *TGFβ*, and *COX2*) have been demonstrated to be upregulated by STAT3 [45]. Apart from the upregulation of prosurvival proteins, STAT3 can potentially decrease the expression of growth suppressor transcription factors, including *p53* and *nedin* [46].

Furthermore, inconsistent chemotherapeutic activity is caused by chemoresistance arising from epithelial–mesenchymal transition (EMT) and cancer stem cells (CSCs) [47]. pSTAT3 has been discovered to be a significant contributor to CSCs and EMT, and inhibiting the STAT3 pathway has been shown to revert chemoresistance. To initiate the activation of EMT-related genes, activated STAT3 in the form of pSTAT3 forms a homodimer and is translocated into the nucleus. Numerous downstream STAT3 targets, including ZEB1, TWIST, SNAIL, FOXM1, and SLUG, facilitate the expression of EMT mediators and ABC transporters, resulting in chemoresistance [48,49,50].

## 4. Terpenoids: Modulators of STAT3 Signaling

**Figure 2:**
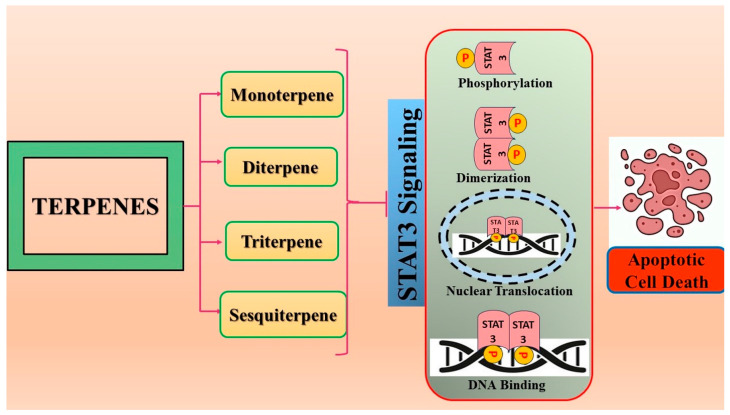
Different terpene classes, including monoterpene, diterpene, triterpene, and sesquiterpene, target STAT3 signaling pathways by directly or indirectly influencing the phosphorylation status of STAT3 protein in various cancer cells.

Phytochemicals harm cancer cells via various methods, including signaling pathway alteration and the initiation of apoptosis. Anti-cancer drugs work by preventing the emergence of carcinogenic species and carcinogens from interacting with cells, which delays the development of tumors [51,52]. The signaling cascades most strongly linked to tumor progression are mitogen-activated protein kinase (MAPK), NF-kB, and activator of transcription proteins (STAT). The metabolic pathways in different types of cancer are controlled by modulating the signaling pathways, which can be overactivated or inhibited. In addition to promoting angiogenesis, enhanced glycolysis, and metastasis, the modifications further contribute to the development and spread of cancer [53]. For the treatment of malignancies, signaling pathways and modified enzymes and factors are significant targets to block or stimulate [54]. Terpenoids are among the bioactive phytocompounds shown to inhibit the JAK/STAT pathway through various methods. There are multiple sites of action in the JAK/STAT pathway that phytochemicals can target [55,56]. These phytochemicals can inhibit this signaling pathway by lowering the quantities of growth hormones or cytokines that activate the JAK/STAT protein [57]. Terpenes can also function by inhibiting JAK phosphorylation before STAT activation. It is possible to regulate JAK/STAT signaling by blocking the translocation of STAT dimer from the cytoplasm into the nucleus and STAT dimerization. The ultimate purpose of the signaling pathway is to prevent STAT-DNA binding, which directly suppresses JAK/STAT-regulated gene transcription [58,59]. This group of phytoconstituents can block the JAK/STAT pathway by preventing STAT3 from being phosphorylated. The phosphorylation of JAK1, JAK2, and c-Src in diverse cancer cell lines was also suppressed by various terpenoids [60] (Table 1) (Figure 2). Subsequent sections address the function of different terpenoids in the JAK/STAT signaling pathway in different types of cancer. In the following sections, we examine the experimental evidence for the ability of various terpenoids derived from multiple plants to modulate the JAK/STAT system in preclinical cancer models.

### 4.1. Monoterpene

#### Thymoquinone

Thymoquinone (TQ), an ingredient obtained from the medicinal plant *Nigella sativa*, has been used in medicine for almost 2000 years. A phytochemical found in black seed (*Nigella sativa*), thymoquinone is widely utilized in traditional medicine in the Middle East, the Mediterranean, South Asia, and Africa. Recent research has discovered that TQ has an adverse effect against many cancer subtypes and suppressed cellular growth in several cancer cell lines, including breast, colon, larynx, lung, myeloblastic leukemia, osteosarcoma, ovary, and pancreatic [126,127,128]. According to a study by Zhu et al., TQ inhibited the expression of multiple STAT3-regulated genes in gastric cancer HGC27 cells, including proliferative (*cyclin D1*), anti-apoptotic (*Bcl-2*, *survivin*), and angiogenic (*VEGF*) gene products [61]. Stimulation of colon cancer cells with TQ reduced nuclear localization, constitutive phosphorylation, and reporter gene activity of STAT3. TQ increased the expression of the cell cycle inhibitory proteins p27 and p21 and decreased the expression of STAT3 target gene products, including *survivin*, *c-Myc*, and *cyclin-D1* and *-D2*. TQ treatment reduced the phosphorylation of upstream kinases, including Src kinase, EGFR tyrosine kinase, and Janus-activated kinase-2 (JAK2). Drug-induced tyrosine phosphorylation of JAK2 and Src attenuated EGFR and STAT3 tyrosine phosphorylation, but EGFR tyrosine kinase inhibitor therapy (gefitinib) suppressed STAT3 phosphorylation in HCT116 colon cancer cells without influencing JAK2 or Src phosphorylation [62]. Al-Rawashde et al. [63] explained that TQ significantly reduced cell proliferation and caused apoptosis by downregulating the expression levels of BCR ABL, JAK2, STAT3, STAT5A, and STAT5B targets in K562 leukemia cells. Another study found that in HL60 leukemia cells, TQ-mediated activation of apoptosis and reduced cell proliferation were linked to the downregulation of essential elements of the JAK/STAT (such as JAK2, STAT3, p-STAT3, STAT5, and p-STAT5) and PI3K/AKT (such as PI3K, p-PI3K, AKT, and p-AKT,) signaling pathways [64]. TQ significantly reduced both constitutive and inducible phosphorylation of STAT3 in breast tumor tissue but did not affect STAT5 in breast cancer. Additionally, it reduced the growth of tumors, downregulated downstream target proteins of STAT3 (such as *Jak2*, *Src*, and *cyclin D1*), and boosted caspase-3 and -9 apoptotic activity in breast cancer. Remarkably, the elevation of tumoral microRNA-125a-5p was concomitant with the synergism of doxorubicin anti-neoplastic action that TQ induced [65]. Moreover, TQ’s growth-inhibitory and apoptosis-inducing actions in renal cell carcinoma and melanoma were linked to the suppression of Jak2/STAT3 by downregulating the phosphorylated level of STAT3 [66,67]. Additional research revealed that by preventing the phosphorylation of the upstream kinase Src, TQ reduced the constitutive phosphorylation and DNA binding activity of signal transducer and activator of transcription-3 (STAT3) in skin cancer and myeloma. Furthermore, TQ administration reduced the expression of the STAT3 target gene products survivin and cyclin D1 [68,69].

### 4.2. Diterpene

#### 4.2.1. Andrographolide

*Andrographis paniculate* (Burm.f.) Nees is a widely growing plant revered as a successful herbal remedy in many nations. Andrographolide is a novel diterpene lactone derived from *A. paniculate* [129]. A growing body of research has shown that AD has anti-inflammatory, anti-HIV, anti-influenza virus, and anti-cancer properties both in vivo and in vitro [130]. By directly and highly selectively binding to STAT3, andrographolide controlled autophagy and programmed death-ligand 1 (PD-L1) expression in NSCLC. Proteomics investigation revealed that andrographolide inhibited JAK2/STAT3 signaling by downregulating the phosphorylation status of STAT3, which in turn decreased the expression of tumor PD-L1 in NSCLC [70]. An additional investigation revealed a correlation between the constitutive activation level of STAT3 and the cancer cell’s ability to withstand doxorubicin-induced apoptosis. The short-term MTT assay and the long-term colony formation experiment revealed that andrographolide significantly increased doxorubicin-induced cell death in cancer cells, showing that andrographolide increases cancer cell sensitivity to doxorubicin primarily through STAT3 inhibition. Mechanistically, andrographolide inhibited IL-6-mediated STAT3 activation and concomitant nuclear transport, which was further associated through the repressed expression of Jak1/2 as well as the interaction between STAT3 and gp130 [71].

#### 4.2.2. Cryptotanshinone

One of the main tanshinones identified from the well-known Chinese herb *Salvia miltiorrhiza* Bunge is called cryptotanshinone (CPT), and it is utilized extensively in conventional and modern medicine [131]. Numerous studies have suggested that CPT may limit the growth and metastasis of malignancies in vivo and the cell growth of cancer cells in vitro [132]. Numerous in vitro tests also revealed various ways in which CPT suppresses tumors. Overexpression of STAT3 has been linked to cancer, and CPT treatment slows the growth of cancer by blocking STAT3 by selectively decreasing STAT3 Tyr705 phosphorylation and its downstream target proteins, which include Bcl-xL, survivin, and cyclin D1 in prostate cancer [72]. Furthermore, ovarian cancer cells treated with CPT showed a significant reduction in lactate generation and glucose uptake. The mechanism by which CPT exhibited its anti-tumor impact involved focusing on the STAT3/SIRT3/HIF-1α signaling cascade both in vitro and in vivo. This pathway may be restored by introducing SIRT3 shRNA into ovarian cancer cells [73]. Ge et al. showed that by decreasing the activity of STAT3 and numerous upstream regulatory signaling pathways, CPT dramatically caused cell death and cell cycle arrest of the BxPC 3 human pancreatic cancer cells [74]. In esophageal cancer cells, CPT also reduced the expression of STAT3 and the activation of STAT3 mediated by IL-6. The mechanism behind the stimulation of apoptosis and prevention of cell proliferation in esophageal cancer cells has been attributed to a CPT-mediated downregulation of STAT3 [75]. Furthermore, cryptotanshinone significantly inhibited cancer cell proliferation, promoting cell death by reducing STAT3 phosphorylation at Tyr705 and impeding nuclear translocation. Cell proliferation was repressed due to the coordinated downregulation of P-AKT, cyclin D1, C-MYC, MEKK2, survivin, and HGF, which halted cell cycle progression at the G0/G1 phase [76]. Some studies have shown that CPT has a growth-inhibiting impact on glioma cells. CPT reduced the nuclear translocation of STAT3 and decreased the phosphorylation of Tyr705 but not Ser727. Cell cycle progression was severely halted in the G1/G0 phase after the STAT3-regulated proteins cyclin D1 and survivin were downregulated [77,78]. Additionally, CPT displayed growth-suppressive effects mediated by STAT3 in chronic myeloid leukemia models [79,80]. These findings suggest that CPT might be a potent antiproliferation medication for managing several types of carcinoma and that STAT3 signaling suppression may play a role in its mode of action.

#### 4.2.3. Oridonin

Oridonin is a naturally occurring terpenoid used in many Chinese medicine formulations. It is derived from *Isodon rubescens* (Hemsl.) H.Hara, a plant used in traditional Chinese herbal medicine. Oridonin has several established anti-cancer properties, including the ability to fight off oral cancer, gastric cancer, nasopharyngeal carcinoma, esophageal cancer, ovarian cancer, leukemia, myeloma, and so forth [133,134,135,136]. Its primary mechanisms include suppression of migration and invasion, induction of apoptosis, inhibition of proliferation, and reversal of drug resistance [137,138]. Oridonin has significantly decreased the migration and invasion of BCPAP and TPC-1 thyroid cancer cells, as demonstrated by the Matrigel invasion experiment, transwell migration assay, and wound healing assay. An increasing body of research suggests that the epithelial–mesenchymal transition is related to the JAK2 (Janus kinase-2)/STAT3 (signal transducer and activator of transcription 3) signaling pathway. As predicted, when oridonin was administered to thyroid cancer TPC-1 and BCPAP cells, the protein levels of phosphorylated-JAK2 and phosphorylated-STAT3 were significantly decreased and also prevented EMT in these cells [81]. In vitro research using human nasopharyngeal cancer cell lines (CNE-2Z and HNE-1) demonstrated that oridonin markedly reduced cell invasion and migration. Western blotting results later showed that oridonin administration decreased the phosphorylation levels of AKT and STAT3. The findings are consistent with the hypothesis that decreased AKT and STAT3 protein activity is linked to oridonin’s anti-metastatic action [82]. Additionally, oridonin induced apoptosis and reduced the growth of U2OS osteosarcoma cells [83]. As demonstrated by DAPI staining and reduced expression of antiapoptotic proteins, the antiproliferative effects were mostly caused by the induction of apoptosis. Additionally, it was shown that oridonin significantly reduced the expression of MMP-2, -3, and -9 in osteosarcoma cells and, in addition, reduced the level of phosphorylation of p-STAT3 (Tyr 705) and p-STAT3 (Ser 727) [83].

### 4.3. Triterpene

#### 4.3.1. Brusatol

Brusatol (Bru), a substance initially identified and extracted from *Brucea sumatrana* seeds in 1968, has long been used to treat amebiasis, malaria, and dysenteric illnesses. With the rapid advancement of medical chemistry, Bru has been reported to be a potent antitumor agent in several types of malignant cells, including lung cancer, pancreatic cancer, colorectal cancer, and leukemia [139]. Hall et al. (1979) revealed that Bru had a potent suppressive impact on tumor cell metabolism and proliferation of lymphocytic leukemia cells by significantly reducing c-myc synthesis [140]. The cell viability, migration, and invasion were all significantly reduced by Bru in Hep-2 laryngeal cancer cells. Bru also blocked Hep-2 cells in the S phase, resulting in cell death in Hep-2 laryngeal cancer cells. Bru further decreased the expression levels of markers related to the epithelial–mesenchymal transition (EMT). Western blotting data demonstrated that Bru could reduce JAK2/STAT3 protein level and phosphorylation status. The anti-tumor action of Bru on Hep-2 cells in vitro was indicated by in vivo tests, where Bru showed no observable toxicity and inhibited the growth of xenograft laryngeal tumors [84]. Bru-induced head and neck squamous cell carcinoma growth was similarly attributed to the disruption of the activation of STAT3 and upstream kinases such as JAK1, JAK2, and Src [85]. It also decreased nuclear STAT3 levels and the protein’s capacity to bind DNA [85]. Bru was shown to reduce proliferation and cause apoptosis in PATU-8988 and PANC-1 pancreatic cancer cells by lowering Bcl-2 expression and enhancing Bax and cleaved Caspase-3 expression. Possible pro-apoptotic signaling pathways are linked to the repression of NF-kB, STAT3, JNK, and p38 MAPK stimulation in pancreatic cancer cells [86]. In hepatocellular carcinoma HCCLM3 cell line and tumor tissues, Bru therapy decreased the levels of many mesenchymal markers while upregulating the expression level of epithelial markers such as occludin and E-cadherin. Furthermore, a decrease in the expression of the transcription factors Twist and Snail was noted. Because STAT3 signaling can control the expression of these two factors, the modulation of this pathway by Bru was also studied [87]. Bru inhibited the phosphorylation of STAT3Y705, while STAT3 knockdown with siRNA resulted in the restoration of epithelial markers. Bru (1 mg/kg) significantly reduced tumor burden in an orthotopic mouse model of liver cancer while decreasing lung metastasis [87].

#### 4.3.2. Betulinic Acid

A pentacyclic triterpene of the lupane class, betulinic acid shares several biological properties with other triterpenoids, most notably anticancer properties. Recent studies on the cytotoxic and anticancer properties of betulinic acid have demonstrated that it promotes apoptosis in cancer cells while enhancing apoptosis throughout the mitochondrial route in healthy cells without harming them [141,142]. In multiple myeloma cells, betulinic acid suppressed constitutive stimulation of STAT3, Src kinase, JAK1, and JAK2. The downregulation of STAT3 activation caused by betulinic acid was reversed by pervanadate, indicating the participation of a protein tyrosine phosphatase (PTP). Moreover, betulinic acid increased PTP *SHP-1* expression, and the inhibition of STAT3 activation by betulinic acid was eliminated by hindering the *SHP-1* gene, preventing betulinic acid-induced apoptosis. Additionally, betulinic acid inhibited the expression of genes that are controlled by STAT3, including *cyclin D1*, *survivin*, *bcl-xL*, and *bcl-2.* A boost in the population of sub-G1 cells and an increase in caspase-3-mediated PARP cleavage suggested a correlation with an increase in apoptosis. In agreement with these findings, betulinic acid-induced apoptosis was considerably attenuated by constitutive active STAT3 overexpression [88]. According to Shin et al. [89], betulinic acid inhibited the phosphorylation level, DNA binding property, and nuclear accumulation of STAT3 in PC-3 prostate cancer cells caused by hypoxia. Furthermore, betulinic acid dramatically decreased cellular and secreted amounts of VEGF, a major angiogenic factor and a target gene of STAT3 produced by hypoxia. Notably, the chromatin immunoprecipitation (ChiP) experiment showed that BA prevented STAT3 and HIF-1α from attaching to the VEGF promoter. Moreover, BA therapy under hypoxia significantly decreased VEGF production, which was successfully increased by siRNA transfection-mediated STAT3 suppression.

#### 4.3.3. Celastrol

Celastrol is a pentacyclic triterpenoid obtained from the traditional Chinese medicinal *Tripterygium wilfordii* and exhibits various pharmacological properties. Celastrol in particular has been shown in modern pharmacological studies to have significant broad-spectrum anticancer activities in the treatment of a variety of cancers, including lung cancer, liver cancer, colorectal cancer, hematological malignancies, gastric cancer, prostate cancer, renal cell carcinoma, breast cancer, osteosarcoma, glioma, cervical cancer, and ovarian cancer [143]. Celastrol suppressed both constitutive and stimulated STAT3 activation in hepatocellular carcinoma, and this inhibition was mediated by inhibiting the expression of upstream kinase c-Src and Janus-activated kinase-1 and -2. Celastrol inhibited STAT3 activity, suppressing several gene products involved in proliferation, survival, and angiogenesis such as *cyclin D1*, *Bcl-2*, *Bcl-xL*, *survivin*, and *VEGF*. Ultimately, celastrol prevented the formation of human HCC xenograft tumors in athymic nu/nu mice and reduced STAT3 activation in tumor tissues when administered intraperitoneally without causing any adverse effects [90]. Overall, these findings show that celastrol shows antiproliferative and proapoptotic effects in HCC by suppressing STAT3 signaling both in vitro and in vivo. Zhao et al. [91] demonstrated that celastrol inhibited the growth, proliferation, and metastasis of NSCLC cells. Celastrol caused a substantial increase in intracellular ROS, which activated the ER stress pathway, inhibited the P-STAT3 pathway, and ultimately resulted in cell death. These effects were countered by pre-treatment with N-acetyl-L-cysteine (NAC). Celastrol’s tumor suppressive effect was also demonstrated in an in vivo lung cancer model of HL60 cells. Furthermore, Kannaiyan and colleagues [92] revealed that celastrol suppressed the proliferation of multiple myeloma (MM) cell lines. It improved thalidomide and bortezomib’s apoptotic effects synergistically. Celastrol’s effects were mediated via phosphorylation of both p65 and IκBα, as well as constitutively active NF-κB produced by inhibition of IκBα kinase activity. Additionally, celastrol reduced the constitutive and IL6-induced stimulation of STAT3 in MM, which resulted in apoptosis, as demonstrated by an increase in the sub-G1-phase cell accumulation, pro-apoptotic protein expression, and caspase-3 activity.

#### 4.3.4. Cucurbitacin B

Cucurbitacin B, or CuB, is a steroid with a unique bitter taste and cytotoxic characteristics found in plants from the *Cucurbitaceae* family and other plant families like Brassicaceae. CuB is a naturally occurring triterpenoid from the Cucurbitaceae family of plants. It has been discovered that there are more than 18 different types of cucurbitacin, with cucurbitacin B being a common kind [144]. CuB has been shown to activate apoptosis in various cancer types via the signaling pathways of Wnt/β-catenin, JAK/STAT, NF-κB, PI3K/AKT, and MAPK/ERK [145,146]. Cucurbitacin B induced growth inhibition and apoptosis in pancreatic cancer cells in a dose- and time-responsive manner [93]. This was connected to decreased expression of cyclin A, cyclin B1, and Bcl-XL, with subsequent activation of the caspase cascade; suppression of active JAK2, STAT3, and STAT5; and elevated levels of p21WAF1 even in cells without functioning p53. Notably, gemcitabine’s antiproliferative effects on pancreatic cancer cells were enhanced by the synergistic interaction of gemcitabine (pyrimidine nucleoside analog) and CuB. Additionally, through JAK2/STAT3 downregulation, CuB reduced the size of pancreatic cancer xenografts in athymic nude mice compared to controls without apparent drug toxicities. In a different study, the anticancer potential of CuB was demonstrated in PANC-1 pancreatic cancer cells through the suppression of Bcl-2, survival expression, and STAT3 activation [94]. According to Yar Saglam et al., CuB, both by itself and in conjunction with gefitinib (a selective inhibitor of EGFR), significantly inhibited the proliferation of colorectal cancer cells and triggered apoptosis. CuB administration alone or coupled with gefitinib reduced cyclin D1, pSTAT3 (Tyr705), pEGFR, Bcl-2, and BCL2L1 levels [95]. Moreover, it was found that in HepG2 hepatocellular carcinoma cells, cucurbitacin B reduced cell viability, induced cell cycle arrest, and formed apoptotic bodies. Protein expression analysis revealed that CuB decreased the expression of Bcl-2 and reduced the phosphorylation of STAT3. CuB also suppressed the growth of a HepG2 tumor in nude mice [96]. Subsequent research revealed that CuB markedly reduced gastric cancer cell growth while inducing apoptosis and cell cycle arrest. The expression of STAT3 and downstream targets, including cyclin D1, c-Myc, and Bcl-xL, markedly decreased with CuB therapy. CuB combined with cisplatin increased gastric cancer cell cytotoxicity, probably due to enhanced reduction in tyrosine phosphorylation of STAT3 (TYR-705). Moreover, the therapeutic efficacy of CuB in vivo was validated using a xenograft mouse model [97,98].

#### 4.3.5. Cucurbitacin E

Cucurbitacin E (CuE), an oxygenated tetracyclic triterpenoid, is prevalent in the fruit pulps of *Citrullus colocynthis*. CuE has been shown to exhibit significant biological activities, such as antiviral, antitumor, and anti-inflammatory properties [147]. It is a type of triterpene that exhibits strong anticancer properties in many cancer cells [148]. Cucurbitacin E has induced dose- and time-dependent apoptosis, cell cycle arrest, and growth suppression in pancreatic cancer cells [99]. According to additional findings from Western blotting, cucurbitacin E therapy can decrease STAT3 phosphorylation while upregulating p53 expression in PANC-1 pancreatic cancer cells.

In the event of prostate cancer, on the other hand, CuE dramatically suppressed downstream protein kinases, including extracellular signal-regulated kinase and p38 mitogen-activated protein kinases, and blocked the vascular endothelial growth factor receptor (VEGFR) 2-mediated Janus kinase (Jak) 2-signal transducer and activator of transcription (STAT) 3 signaling pathway [100]. Additional research on bladder cancer cells revealed that cucurbitacin E-induced G2/M arrest might be associated with downregulation of pSTAT3, cyclin-dependent kinase 1 (CDK1), and cyclin B in human bladder cancer T24 Cells [101].

#### 4.3.6. Cucurbitacin I

The natural tetracyclic triterpenoid cucurbitacin I (CuI), which is derived from the Cucurbitaceae and Cruciferae families, has been utilized in traditional medicine for its antipyretic, analgesic, anti-inflammatory, and antibacterial properties. According to recent results, CuI, one of 12 cucurbitacin derivatives, has a powerful anticancer effect against different cancer cells, such as lung cancer, breast cancer, prostate cancer, cervical cancer, colon cancer, gastric cancer, and hepatocellular carcinoma [149]. Numerous studies have revealed that STAT3 is an indirect molecular target of CuI’s anticarcinogenic action [150]. CuI causes cell apoptosis, inhibits the growth of pancreatic cancer, and stops the cell cycle in the G2/M phase [102]. Additionally, the compound significantly slowed down cell invasion potential and hindered the development of pancreatic tumor xenografts in nude mice. Furthermore, CuI-induced suppression of pancreatic cancer cell growth appears to include JAK2/STAT3 signaling pathway suppression because JAK2/STAT3 activators successfully prevented the inhibition [102]. Furthermore, it was demonstrated that administering CuI to HepG2 hepatocellular carcinoma cells decreases the number of proteins in the PI3K/AKT/mTOR, MAPK, and JAK2/STAT3 cascades. In varying doses, CuI also reduced the expression of the genes for *MAPK*, *STAT3*, *mTOR*, *JAK2*, and *Akt* [103]. Ni et al. found that CuI suppresses ERK activation and the downstream phosphorylation level of mTOR and STAT3, which may cause reduced viability of A549 lung cancer cells, impeded colony formation, and induced apoptosis [104]. Another study that examined the anticancer potential of various cucurbitacin compounds in different cancer models found that CuI inhibited the activation of STAT3 and JAK2 in A549 lung cancer cells [105]. Furthermore, cucurbitacin E and B inhibited the activation of both STAT3 and JAK2 but mildly activated apoptosis and suppressed tumor growth in lung cancer. Subsequent analysis revealed that glioblastoma cells treated with CuI had lower levels of phosphorylated-STAT3, accompanied by increased apoptosis and cell cycle arrest. In particular, CuI caused G2/M accumulation in glioblastoma cells by downregulating the downstream targets of STAT3 such as cyclin B1 and cdc2 levels [106].

Furthermore, this compound was found to lower the levels of phosphorylated STAT3 and Janus kinase-3 (JAK3), which may be a significant factor in the reduction of cell viability, cell cycle arrest, and activation of apoptosis in lymphomas. Alongside these modifications, there were notable decreases in several other downstream targets of STAT3, such as cyclin D3, mcl-1, bcl-2, and bcl-xL [107].

#### 4.3.7. Ursolic Acid

The cyclosqualenoid family contains ursolic acid (UA), a ursane-type pentacyclic triterpene acid widely found in the leaves and berries of naturally occurring medicinal plants, including *Vaccinium macrocarpon* Air. (cranberry), *Arctostaphylos uva-ursi* (L.) Spreng (bearberry), *Calluna vulgaris*, *Ocimum sanctum*, and many more. Recent research has shown evidence of ursolic acid’s therapeutic benefits in various human disorders, including different kinds of malignancies caused by inflammation [151].

In A549 and H460 lung cancer cells, UA was explicitly recognized as suppressing STAT3 activity. Additionally, it prevented the growth of tumor spheres, migration, invasion, and angiogenesis. UA efficacy involves UA binding to the epidermal growth factor receptor (EGFR), lowering pEGFR levels, and blocking the downstream JAK2/STAT3 pathway. Moreover, UA decreased the expressions of MMPs, VEGF, and PD-L1, as well as the formation of STAT3/MMP2 and STAT3/PD-L1 complexes [108]. Additionally, the effects of UA on inhibiting the nuclear translocation of STAT3 in colorectal cancer cells were assessed, and it was found that in HCT116 and HT29 cells, UA substantially decreased the expression of JAK2 and STAT3 [109]. Moreover, it is commonly recognized that IL-6 treatment results in the phosphorylation of STAT3 [152]; consequently, the authors investigated whether UA could prevent the STAT3 phosphorylation that IL-6 produced. UA significantly decreased P-STAT3, which produces interleukin-6 (IL-6) in hepatocellular cancer cells. In these cell lines, UA reduced the expression of STAT3’s downstream target genes, including *Bcl-2*, *Bcl-xl*, and *survivin*. UA also inhibited colony formation, cell migration, and viability in liver cancer cells. In vivo, oral daily administration of UA inhibited both the development of HEPG2 tumors and STAT3 phosphorylation [110]. In the dorsolateral prostate (DLP) tissues of a TRAMP mouse model of prostate cancer, UA inhibited the activation of many pro-inflammatory mediators, such as NF-κB, STAT3, AKT, and IKKα/β phosphorylation. This was linked with a decrease in blood levels of TNF-α and IL-6. Furthermore, immunohistochemical analysis of tumor tissue sections showed that UA dramatically up-regulated the levels of caspase-3 as well as substantially downregulated the expression of cyclin D1 and COX-2 [111].

### 4.4. Sesquiterpene

#### 4.4.1. Alantolactone

One of the most significant sesquiterpenes, alantolactone (ALT), is obtained in substantial quantities from the root of *Inula helenium* (elecampane). Some plants, including Inula species *Aucklandia lappa*, *Rudbeckia subtomentosa*, and *Radix inulae*, can also provide ALT, in addition to *Inula helenium* [153]. According to in vitro research [154], this substance prevents the growth of different cancer cells, including those found in breast, stomach, colon, pancreatic, and squamous cell lung malignancies. An increasing amount of data from recent years has indicated that ALT demonstrates anticancer potential by targeting the cell cycle, cell proliferation, invasion and metastasis, and the activation of apoptosis, as well as by blocking numerous signaling pathways implicated in the progression of cancer [155]. ALT inhibited the STAT3 phosphorylation and signaling pathway in prostate cancer cells after 72 h. Additionally, by upregulating the expression of p53 and downregulating the expression of SOX2, Oct-4, Nanog, CD133, and CD44, ALT was able to modify the stemness of prostate cancer cells [112]. Ahmad et al. [113] showed that, after suppressing the production of STAT3 and survivin, ALT reduced cell viability and enhanced cell death and apoptosis in human leukemia THP-1 cells. ALT was also shown to demonstrate potential efficacy against triple-negative breast cancer MDA-MB-231 cells by decreasing the STAT3 signaling pathway [114]. Alantolactone efficiently inhibited both constitutive and inducible STAT3 activation at tyrosine 705. The nucleus translocation of STAT3, its DNA-binding activity, and the expression of STAT3 target genes were all reduced by alantolactone. ALT significantly inhibited constitutively activated STAT3 in pancreatic cancer cells while having minimal effect on the EGFR pathway. Additionally, both in vitro and in vivo, the combination of alantolactone and an EGFR inhibitor such as erlotinib or afatinib showed a remarkable combinatorial anticancer effect against pancreatic cancer cells [115].

#### 4.4.2. β-Caryophyllene Oxide

Natural bicyclic sesquiterpenes such as β-caryophyllene (BCP) and β-caryophyllene oxide (BCPO) are found in various plants worldwide, such as basil (*Ocimum* spp.), cinnamon (*Cinnamomum* spp.), black pepper (*Piper nigrum* L.), cloves (*Syzygium aromaticum*), cannabis (*Cannabis sativa* L.), and lavender (*Lavandula angustifolia*). Significant anticancer effects are possessed by both BCP and BCPO (BCP(O)), which inhibit the growth and multiplication of different cancer cells in vitro, such as cervical cancer HeLa cells, liver cancer HepG2 cells, leukemia AGS cells, gastric cancer SNU-1 cells, and stomach cancer SNU-16 cells [156]. CPO inhibited constitutive STAT3 stimulation in multiple myeloma, breast, and prostate cancer cell lines, with MM cells exhibiting significant dose- and time-dependent effects. The CPO-mediated STAT3 suppression was accomplished by blocking the activation of JAK1/2 and c-Src, two upstream kinases. Additionally, the down-regulation of STAT3 caused by CPO was reversed via vanadate therapy, indicating the participation of tyrosine phosphatase [116]. The authors observed that constitutive STAT3 activation was down-regulated in correlation with the tyrosine phosphatase SHP-1 expression that CPO promoted. Interestingly, CPO’s capacity to block STAT3 activation was eliminated with siRNA ablation of the SHP-1 gene. By blocking STAT3 activation, CPO reduced the ability of MM1.5 multiple myeloma tumor cell lines to grow, triggering apoptosis. The results indicate that CPO is a novel STAT3 signaling cascade blocker, which opens up an array of therapeutic options for various malignancies that have constitutively activated STAT3 [116].

#### 4.4.3. Dihydroartemisinin

Artemisinin, a plant-derived antimalarial medicine, has a semisynthetic derivative called dihydroartemisinin (DHA). It is substantially more water soluble and has more antimalarial activity than other artemisinin derivatives [157]. According to recent research, DHA demonstrates anticancer effects by causing cell death, altering the cell cycle, and preventing tumor angiogenesis. DHA adversely affects several oncological signaling pathways, such as the JAK/STAT, Wnt/β-catenin, MAPK/ERK, and translationally controlled tumor protein (TCTP) cascades [158]. DHA suppressed breast cancer cell growth and promoted apoptosis in DDP (cisplatin)-resistant cells by inhibiting STAT3/DDA1 signaling. DDA1 (DET1- and DDB1-associated 1) knockdown increased DDP-resistant cell death, suppressed cell proliferation, enhanced G0/G1 phase arrest, and decreased cyclin D1 expression. Also, by targeting DDA1, STAT3 knockdown inhibited the growth of DDP-resistant cells, causing apoptosis and G0/G1 cell cycle arrest [117]. Additionally, DHA efficiently inhibited the phosphorylation of STAT3 and the inactivation of STAT3, leading to the downregulation of Mcl-1 and survivin, which improved the chemosensitivity of ABT-263 in lung cancer cells [118]. Subsequently, DHA and ABT-263 cotreatment substantially decreased xenograft development in nude mice. Jia et al. [119] also noticed that DHA exclusively blocked JAK2/STAT3 signaling in HNSCC head and neck cancer cells, exhibiting significant and specific inhibitory effects on STAT3 phosphorylation. Furthermore, DHA substantially decreased HNSCC growth in vivo (xenograft tumor model of Cal-27 cells) and in vitro (Fadu, Cal-27 and Hep-2), possibly by reducing cell migration and inducing death. Additionally, combined treatment of DHA and cisplatin decreased the growth of HNSCC tumors. A report by Wang et al. showed that DHA suppressed Jak2/STAT3 signaling, which in turn increased cell death and altered the expression of several apoptotic-related proteins in colon cancer cells [120]. Furthermore, DHA reduced the activity of the IL-6/STAT3 signaling pathway and prevented the downregulation of STAT3 and β-catenin protein expression mediated by miR-130b-3p. Moreover, the authors revealed that miR-130b-3p demonstrates direct targeting capability for STAT3, which reduces invasion by LSCC (laryngeal squamous cell carcinoma). DHA can prevent IL-6-induced EMT and invasion in LSCC by increasing miR-130b-3p expression, which reduces activation of the IL-6/STAT3 and β-catenin signaling pathways [121]. DHA also inhibited B16F10 melanoma cell growth in vitro as well as in vivo in BALB/C mice. In addition, DHA dose-dependently prevented melanoma invasion, migration, and colony formation. In the tumor and spleen, DHA promoted the level of expression of IFN-γ and decreased the levels of IL-10 and IL-6. Furthermore, by controlling the STAT3 pathway, DHA treatment significantly accelerated the mitochondrial apoptosis of melanoma [122].

#### 4.4.4. Parthenolide

Parthenolide (PN) is a sesquiterpene lactone obtained from feverfew (*Tanacetum parthenium*), a medicinal herb. It has an α-methylene-γ-lactone ring and an epoxide group that can interact with the nucleophilic sites of biological molecules. Due to its comprehensive evaluation, PN has been used as an herbal medicine for millennia because of its anti-inflammatory and anti-migraine qualities. The potential of PN as an anticancer agent has garnered significant interest recently [159]. PN has been shown to suppress the STAT3 signaling pathway in SGC-7901/DPP gastric cancer cells, resulting in apoptosis and enhanced drug chemosensitivity. PN treatment inhibited cancer cell growth by inhibiting STAT3-mediated production of Bax, Bcl-2, Bcl-xL, and cyclin D1 [123]. PN was also found to be a potent inhibitor of JAKs in HepG2 liver cancer cells and MDA-MB-231 breast cancer cells. It inhibited the kinase activity of JAK2 by covalently altering its Cys178, Cys243, Cys335, and Cys480 residues. It also had comparable interactions with other JAKs. Moreover, PN suppressed the IL-6-induced migration of cancer cells and specifically halted the development of tumors expressing a constitutively active STAT3 [124].

#### 4.4.5. γ-Tocotrienol

γ-Tocotrienol, a vitamin E superfamily member obtained from rice bran and palm oil, has garnered significant interest due to its putative anticarcinogenic and antiproliferative properties [160]. γ-Tocotrienol reduced both constitutive and inducible STAT3 activation while having no effect on STAT5. γ-Tocotrienol also prevented the activation of JAK1, JAK2, and Src, which are involved in the activation of STAT3. The down-regulation of STAT3 mediated by γ-tocotrienol was reversed by pervanadate, indicating the participation of a protein tyrosine phosphatase. In fact, γ-tocotrienol promoted the expression of a protein tyrosine phosphatase SHP-1, and its capacity to prevent STAT3 activation was eliminated upon SHP-1 gene deletion via small interfering RNA. Additionally, γ-tocotrienol suppressed the expression of gene products controlled by STAT3, such as cyclin D1, Bcl-2, Bcl-xL, survivin, Mcl-1, and VEGF. Lastly, γ-tocotrienol reduced proliferation, caused apoptosis, and significantly increased the apoptotic effects of chemotherapeutic drugs (paclitaxel and doxorubicin) in hepatocellular carcinoma HepG2 cells [125].

## 5. Conclusions and Future Perspectives

Although STAT3 expression is tightly controlled in normal cells, it is constitutively activated in various malignancies. The abnormal activation of STAT3 promotes tumor metastasis by promoting tumor cell proliferation, angiogenesis, migration, and invasion. Furthermore, evading immune surveillance is significantly aided by the activation of STAT3 signaling. Since numerous types of cancer are linked to STAT3 expression and function, natural phytocompounds have made this protein their main therapeutic target. Several epidemiological and preclinical investigations have explored the relationship between dietary terpenoids and the suppression of the transcription factor STAT3 in various cancer cell lines. Several terpenoid family chemicals have demonstrated strong evidence of their chemopreventive and chemoprotective properties. Among them, some compounds directly target STAT3, whereas others indirectly reduce its function. Furthermore, malignant cells with abnormal STAT3 activation exhibit increased cellular invasion, migration, differentiation, and growth. Natural terpenes have been thoroughly studied for their ability to inhibit the signaling arrays of unregulated STAT3 in cancer cells. With regard to these sixteen drugs, there is still a lack of information about antitumor potential by targeting the STAT3 signaling cascade in animal models, particularly in humans. In order to corroborate and clinically establish the chemopreventive nature of terpenoids, it can be recommended for additional studies including both humans and animals to be carried out. Based on this review, we suggest further research to identify more natural polyphenol candidates that can efficiently target STAT3. We predict that this review will provide new insight into future research on plant-derived terpenoids to treat various cancers by targeting STAT3 pathways.

## Figures and Tables

**Table 1:** Different classes of terpenes exhibiting their anticancer potential by downregulating key molecules of STAT3 signaling pathway.

Class	Phytochemical	Cancer	Model	Molecular Target	Mechanism	References
Monoterpene	Thymoquinone 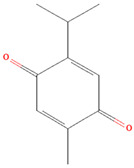	Gastric cancer	HGC27, BGC823, SGC7901 cells; xenograft tumor mouse model	JAK2, STAT3, Src	Reduced tumor growth, induced apoptosis	[61]
		Colon cancer	HCT116 cells	JAK2, STAT3, Src	Inhibited cell proliferation, induced apoptosis	[62]
		Leukemia	K562 cells	JAK2, STAT3, STAT5	Inhibited cell proliferation, induced apoptosis	[63]
		Acute myeloid leukemia	HL60 cells	c-Myc, PI3K, AKT, mTOR, JAK2, STAT3, STAT5a, STAT5b	Inhibited cell proliferation, induced apoptosis	[64]
		Breast cancer	Mice bearing solid Ehrlich tumors	STAT3, caspase-3/9	Attenuated tumor growth, induced apoptosis, chemosensitivity	[65]
		Melanoma	SK-MEL-28 cells, SK-MEL-28 tumor xenografts	Jak2, STAT3	Induced apoptotic cell death	[66]
		Renal cell carcinoma	Caki-1 cells, tumor xenograft mice	Jak2, STAT3, cyclin D2	Attenuated tumor growth, induced apoptosis	[67]
		Skin cancer	A431 cells, tumor xenograft mice	STAT3, Src, cyclin D1	Attenuated tumor growth, induced apoptosis	[68]
		Myeloma	U266 and RPMI 8226 cells	STAT3, c-Src, JAK2	Attenuated cell growth, induced apoptosis, G1 cell cycle arrest, chemosensitivity	[69]
Diterpene	Andrographolide 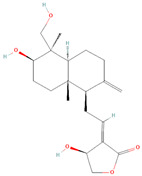	Non-small-cell lung cancer (NSCLC)	H1975 and H1299 cell lines	STAT3, PARP, PD-L1, P62	Inducted autophagy, antitumor immune response	[70]
		Multiple cancer cell types	HCT116, MDA-MB-231, HepG2, HeLa, TPC-1	STAT3, Bcl-xl, cyclin D1	Induced cell death, chemosensitivity	[71]
	Cryptotanshinone 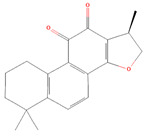	Prostate cancer	DU145 cells	JAK2, STAT3	Retarded cell proliferation, induced apoptosis	[72]
		Ovarian cancer	Hey and A2780 cells	HIF- 1α, STAT3	Retarded cell proliferation and glucose metabolism	[73]
		Pancreatic cancer	BxPC-3 cells	JAK2, STAT3, mTOR, Akt	Retarded cell proliferation, induced apoptosis	[74]
		Esophageal cancer	EC109 and CAES17 cells, athymic nude mice	JAK2, STAT3	Retarded cell proliferation, induced apoptosis	[75]
		Renal cell carcinoma	A498, 786-O, ACHN	STAT3, cyclin D1, Bcl-2	Retarded cell proliferation, induced apoptosis	[76]
		Glioma	T98G and U87 cells	STAT3, cyclin D1, survivin	Suppressed cell viability, induced cell cycle arrest and apoptosis	[77]
		Glioma	C6, U251, T98G, U87; nude xenograft mice	STAT3, SHP2, cyclin D1	Inhibited cell proliferation	[78]
		Chronic myeloid leukemia	K562-R cells, xenografts in nude mice	STAT3, eIF4E	Inhibited tumor growth, induced apoptosis	[79]
		Chronic myeloid leukemia	K562 cells	STAT3, STAT5	Suppressed key oncogenic proliferation, drug resistance	[80]
	Oridonin 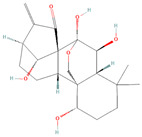	Thyroid cancer	TPC-1 and BCPAP cells	JAK2, STAT3	Inhibited metastatic, angiogenesis, and modulated EMT	[81]
		Nasopharyngeal carcinoma	CNE-2Z and HNE-1 cells	Akt, STAT3	Inhibited metastatic, angiogenesis, and modulated EMT	[82]
		Osteosarcoma	U2OS cells	STAT3, MMP-2, 3, 9	Suppressed cell viability, induced apoptosis	[83]
Triterpene	Brusatol 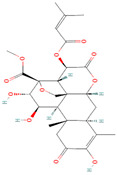	Laryngeal cancer	Hep-2 cells, xenograft laryngeal tumor	JAK2, STAT3	Inhibited viability, migration, and invasion ability	[84]
		Head and neck squamous cell carcinoma	UMSCC 47, UD SCC2, JMAR, Tu167, LN686, FaDu	JAK1, JAK2, STAT3, Src	Reduced cell growth, induced apoptosis	[85]
		Pancreatic cancer	PANC-1 and PATU-8988 cells	NF-κb, Stat3	Reduced cell growth, induced apoptosis	[86]
		Hepatocellular carcinoma	HCCLM3 cell line	STAT3	Inhibited cell migration and invasion	[87]
	Betulinic acid 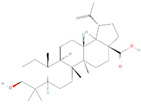	Multiple myeloma	U266 and MM.1S cells	JAK1, JAK2, STAT3, Src	Induced apoptotic cancer cell death	[88]
		Prostate cancer	PC3	HIF-1α, STAT3	Exert anti-angiogenic activity	[89]
	Celastrol 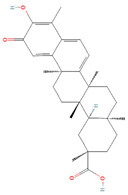	Hepatocellular carcinoma	C3A, HepG2, Hep3B, PLC/PRF5; athymic nu/nu mice	JAK1, JAK2, STAT3, Src	Inhibited cell migration and invasion, induced apoptosis	[90]
		NSCLC	H460, PC-9, H520, BEAS-2B, PC-9 cells; thymic BALB/c nude mice	STAT3, Bcl-2	Reduced cell growth, proliferation, and metastasis	[91]
		Multiple myeloma	U266, RPMI 8226	IκBα kinase, STAT3	Suppressed cell viability, induced cell cycle arrest and apoptosis	[92]
	Cucurbitacin B 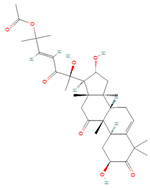	Pancreatic cancer	MiaPaCa-2, AsPC-1	JAK2, STAT3, and STAT5 activation	Cytotoxicity	[93]
		Pancreatic cancer	PANC-1	STAT3	Growth and cell cycle inhibition, induced apoptosis	[94]
		Colorectal cancer	HT-29, HCT-116	STAT3	Growth and cell cycle inhibition, induced apoptosis	[95]
		Hepatocellular carcinoma	HepG2	STAT3	Cell cycle arrest, retarded cell growth	[96]
		Gastric cancer	MKN-45	STAT3	Cell cycle arrest, retarded cell growth	[97]
		Gastric cancer	SGC7901, BGC823, MGC803, MKN74; human gastric cancer xenograft	STAT3, c-Myc, Bcl-xL	Suppressed invasion, induced apoptosis	[98]
	Cucurbitacin E 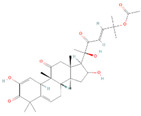	Pancreatic cancer	PANC-1	STAT3	Cell cycle arrest, retarded cell growth	[99]
		Prostate cancer	PC-3, xenograft	STAT3, JAK2	Inhibited angiogenesis, proliferation, survival, and migration	[100]
		Human bladder cancer	T24 cells	STAT3, CDK1, cyclin B	Induced G(2)/M phase arrest and apoptosis	[101]
	Cucurbitacin I 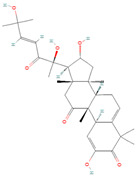	Pancreatic cancer	ASPC-1, BXPC-3, CFPAC-1, SW 1990; orthotopic xenograft mice	STAT3, JAK2	Inhibited proliferation	[102]
		Hepatocellular carcinoma	HepG2 cells	STAT3, JAK2	Induced antiproliferation and G2/M phase of cell cycle	[103]
		Lung cancer	A549 cells	ERK, mTOR, STAT3	Decreased cell viability, inhibited colony formation, induced apoptosis	[104]
		Lung cancer	A549; nude mouse tumor xenograft model	STAT3	Suppressed tumor growth, induced apoptosis	[105]
		Glioblastoma	U251 and A172 cells	STAT3, cyclin B1, cdc2	Decreased cell viability, induced G2/M cell cycle arrest, induced apoptosis	[106]
		Lymphoma	ALK+ ALCL cell lines	STAT3, JAK3, NPM-ALK	Decreased cell viability, induced G2/M cell cycle arrest, induced apoptosis	[107]
	Ursolic Acid 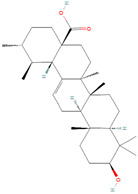	Lung cancer	A549 and H460 cells	STAT3, MMP2, PD-L1, VEGF	Attenuated cell growth, invasion, migration, angiogenesis induced apoptosis, G1 cell cycle arrest, chemosensitivity	[108]
		Colorectal cancer	HCT116 and HT29 cells	STAT3	Induced apoptotic cell death	[109]
		Hepatocellular carcinoma	Hep3B, HEPG2, SSMC-7721, and Huh7 cells; mouse xenograft tumor model	STAT3	Suppressed cell viability, cell migration, and colony formation	[110]
		Prostate cancer	TRAMP mice	NF-κB, STAT3, AKT, IKKα/β	Reduced tumor growth	[111]
Sesquiterpene	Alantolactone 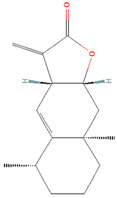	Prostate cancer	PC3	SOX2, Oct-4, Nanog, CD133, CD44, STAT3	Antimetastatic potential	[112]
		Leukemia	THP-1 cells	STAT3, survivin	Decreased cell viability, increased cell death and apoptosis	[113]
		Breast cancer	MDA-MB-231 cells	STAT3, MAPKs, NF-κB	Inhibition of migration, invasion, and adhesion	[114]
		Pancreatic cancer	BxPC-3, AsPC1, and PANC-1 cells	STAT3, Bcl-2	Reduced cell growth, induced cell death	[115]
	β-Caryophyllene oxide 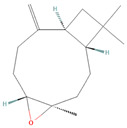	Human multiple myeloma (MM) cell lines, human prostate carcinoma, human breast carcinoma	U266, MM1.S, DU145, MDAMB-231	JAK1, JAK2, STAT3, Src	Reduced cell proliferation and invasion, induced apoptosis	[116]
	Dihydroartemisinin 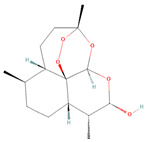	Breast cancer	MDA-MB-231 cells	STAT3, DDA1	Repressed cell proliferation, induced apoptosis	[117]
		Lung cancer	H1975, HCC827, H1650, H3255, A549, H727	STAT3, Mcl-1, survivin	Bax-dependent apoptosis	[118]
		Head and neck Carcinoma	Fadu, Cal-27, and Hep-2 cells; xenograft model	Jak2, STAT3	Apoptosis induction, attenuation of cell migration	[119]
		Colon cancer	HCT116	Jak2, STAT3	Suppressed cell viability, induced apoptosis	[120]
		Laryngeal squamous cell carcinoma	AMC-HN-8 and Tu212 cells	IL-6/STAT3, β-catenin	Repressed EMT and invasion	[121]
		Melanoma	B16F10 cells; BALB/c xenograft mice	STAT3, IL-10, IL-6	Induced apoptotic cell death	[122]
	Parthenolide 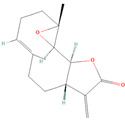	Gastric cancer	SGC-7901/DDP cell	JAK2, STAT3, cyclin D1	Decreased cell viability, induced G1 cell cycle arrest, induced apoptosis	[123]
		Multiple cancer subtypes	HepG2, MDA-MB-231, MDA-MB-468, HCT116, HT-29, Lovo, NCI-H1299, Colo205, BGC, H460, and Du145 cells	JAK1, STAT3,	Suppressed cellular growth and migration	[124]
	γ-Tocotrienol 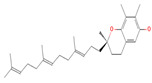	Hepatocellular carcinoma	HepG2, C3A, SNU-387, and PLC/PRF5 cells	STAT3, cyclin D1, Bcl-2, Bcl-xL, survivin, Mcl-1, VEGF	Inhibited proliferation, induced apoptosis, chemosensitivity	[125]
